# Nanoscale precipitates strengthened lanthanum-bearing Mg-3Sn-1Mn alloys through continuous rheo-rolling

**DOI:** 10.1038/srep23154

**Published:** 2016-03-18

**Authors:** R. G. Guan, Y. F. Shen, Z. Y. Zhao, R. D. K. Misra

**Affiliations:** 1 School of Materials Science and Engineering, Northeastern University, Shenyang 110819, P.R. China; 2 State Key Laboratory of Advanced Processing and Recycling of Non-ferrous Metals, Lanzhou University of Technology, Lanzhou 730050, P.R. China; 3 Department of Metallurgical, Materials and Biomedical Engineering, University of Texas at El Paso, TX 79968, USA

## Abstract

We elucidate the effect of lanthanum (La) on the microstructure and mechanical properties of Mg-3Sn-1Mn-*x*La (wt.%) alloy plates processed through continuous rheo-rolling for the first time. At *x* = 0.2 wt.%, La dissolved completely in the α-Mg matrix. As the La content was increased to 0.6 wt.%, a new plate-shaped three-phase compound composed of La_5_Sn_3_, Mg_2_Sn and Mg_17_La_2_ phases was formed with an average length of 380 ± 10 nm and an average width of 110 ± 5 nm. This compound had a pinning effect on the α-Mg grain boundary and on dislocations. With further increase in La-content to 1.0 wt.%, the length of the plate-shaped compound increased to an average length of 560 ± 10 nm, while the width was reduced to 90 ± 5 nm. The particle size of Mg_2_Sn decreased from 100 nm to 50 nm with increase in La-content from 0.2 to 1.4 wt.%. At La content of 1.0 wt.%, the tensile strength and elongation of the alloy was maximum, with 29% and 32% increase, respectively, compared to the Mg-3Sn-1Mn (wt.%) alloy, and 37% and 89% increase, in comparison to the Mg-3Sn-1Mn-0.87 Ce (wt.%) alloy.

Magnesium (Mg) is abundant in natural reserves and its alloys have a number of attractive properties^1^,^2^. Specifically, Mg-alloys have high specific strength and specific stiffness, good damping and shock absorbing capacity, high thermal conductivity, and strong electromagnetic shielding ability^1^,^2^. Thus, Mg alloys are widely used for automotive, aerospace, and packaging applications. However, cold processing of Mg alloys is challenging because Mg has a hexagonal close-packed (HCP) lattice with limited slip systems, making their deformation at low temperature difficult. Another limiting factor that restricts the use of Mg alloys is their low thermal stability leading to deterioration in mechanical properties at high temperatures. The melting temperature of Mg_17_Al_12_ phase in currently used Mg alloys (AZ and AM series) is ~735 K. In a number of structural applications, it is desirable to have high strength and superior corrosion resistance.

Microalloying has been actively pursued to enhance the mechanical properties of Mg-alloys. Certain microalloying elements form strengthening phases in the α-Mg matrix or at the grain boundaries, resulting in solid-solution strengthening and/or precipitation strengthening. These secondary strengthening phases, formed through microalloying, can assist in retaining a high dislocation density in the alloy by promoting the multiplication of dislocations and by pinning them, thereby improving the mechanical properties of Mg-alloys^3^,^4^. Previous research has shown that the addition of Si or Sn in magnesium alloys led to the formation of thermally stable and high-hardness Mg_2_Si or Mg_2_Sn phases, and thus improved the mechanical properties of Mg alloys at room and high temperatures^5^,^6^,^7^,^8^. We selected Sn instead of Si as one of the alloying elements considering the advantages of Mg_2_Sn phase and disadvantages of Mg_2_Si phase. The Mg_2_Sn phase has a high melting temperature of 1051 K, which is significantly higher than 735 K of Mg_17_Al_12_ phase in the currently used AZ series of Mg alloys. Additionally, the Mg_2_Sn phase has a high hardness of 119 HV. The high melting temperature and high hardness of Mg_2_Sn phase makes it an excellent strengthening phase for enhancing mechanical properties and thermal stability^9^,^10^,^11^,^12^. In contrast, the Mg_2_Si phase has a strong tendency to coarsen grains in as-cast Mg-alloys, thereby decreasing strength, reducing casting ability of the alloys, and increasing their brittleness at room temperature. In addition to Sn, Mn is also a beneficial element from the perspective of solid solution strengthening and grain refinement^13^,^14^. Moreover, Bursik *et al*. demonstrated good creep resistance of Mg-*x*Sn-1Mn (*x* = 3 and 5 wt.%) alloys^15^. Shi and Huang *et al*. reported new relationship between the Mg_2_Sn phase and the α-Mg matrix in the Mg-Sn-Mn alloy^16^,^17^.

Rare earth elements play an important role in governing the ultimate properties of Mg alloys. For example, Wei *et al*. studied the creep properties of Mg-Sn-La alloys at high temperatures and observed that Mg–*x*Sn–2La (*x* = 5, 6.5 and 8.5 wt.%) exhibits superior creep properties at high temperatures than Mg-5Sn (wt.%) alloy at 473 K and 25–35 MPa. The main reason for this observation was the presence of rare-earth strengthening phase observed in Mg–*x*Sn–2La (*x* = 5, 6.5 and 8.5 wt.%) alloy^11^. Although La is a beneficial alloying element, the influence of La on the microstructure and mechanical properties of Mg_2_Sn and other phases in Mg-3Sn-1Mn-*x*La (wt.%) alloy, and strengthening mechanisms remain largely unexplored.

Considering that cold processing of Mg alloys is difficult, we explored a novel semi-solid processing, specifically, continuous rheo-rolling, to produce Mg-3Sn-1Mn-*x*La (wt.%) alloy and improve the ability of the alloy to deform. Semi-solid processing has recently attracted significant attention to process magnesium-based alloys^18^. However, extensive studies are required to translate this novel process to industrial applications. Continuous rheo-rolling is a highly efficient semi-solid processing method in which a semi-solid metal composed of a liquid phase and a spherical solid phase is directly rolled following melting. In rheo-rolling, multiple traditional solid processing steps, including ingot casting, solidification, re-heating, and rolling, are integrated into one process, which makes it cost-effective and energy-efficient process. Moreover, rheo-rolling has a higher processing speed for non-ferrous metals than liquid metal roll casting. Using continuous rheo-rolling, Haga (Osaka Institute of Technology, Japan) produced A356 aluminum (Al) alloy plates with superior mechanical properties (tensile strength of 270 MPa) compared to A5052-H34, A6063-T4 and T6^19^. Continuous rheo-rolling effectively addresses the problem of poor deformability of Mg-alloys at room temperature. In this study, rheo-rolling processing with a novel shearing/vibration device for semi-solid metals was used to produce Mg-3Sn-1Mn (wt.%) alloy plates to improve the microstructure and properties of the alloy^20^.

The objective of the study described here is to process Mg-3Sn-1Mn-*x*La (wt.%) alloy plates with La concentration in the range of 0.2 to 1.4 wt.% using continuous rheo-rolling process, and elucidate the effect of La on the microstructure and mechanical properties of Mg-3Sn-1Mn-*x*La (wt.%) alloy plates that has not been previously explored.

## Experimental Procedure

**Figure 1:**
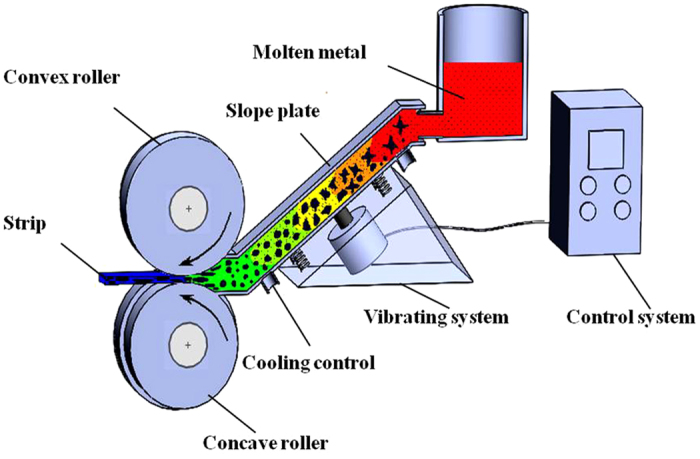
Schematic illustration of a rheo-rolling process in this study.

A self-designed continuous rheo-rolling experimental apparatus was used to conduct the experiments^20^. The roller diameter was 400 mm, the cross-sectional area of the prepared alloy plates was 4 mm × 160 mm, and the maximum processing rate was 22 m/min. Figure 1 shows a schematic configuration of the equipment used in this study^20^. A molten alloy was cast onto the vibrating slope plate to form a high-quality semi-solid metal slurry via flow shear and vibration effects. This semi-solid metal slurry entered directly into the bottom of the width-restricted roll for rheo-rolling. The process has two main advantages: (i) the temperature of the semisolid slurry is significantly lower than the melt in conventional roll casting, resulting in short solidification time and consequently very high rolling speed for the semi-solid alloy; and (ii) the process is expected to be developed as a high-speed semi-solid roll-casting technique. Thus, using this approach, the mechanical properties of the strip can be improved through tailoring of the microstructure.

The Mg-3Sn-1Mn-*x*La (*x* = 0.2, 0.6, 1.0 and 1.4 wt.%) alloys used in this study were prepared using 99.95 wt.% pure Mg, 99.95 wt.% pure Sn, Mg-25La (wt.%) alloy, and Mg-4.38Mn wt.% alloy. The nominal chemical composition (in wt.%) of the alloy was Mg-3% Sn-1% Mn-(0.2, 0.6, 1.0, 1.4%) La, with traces of 0.01% Si, 0.05% Cu, 0.01% Ni, and 0.01% Fe. A resistance furnace (3 kW, SG2-3-9, Shenyang General Furnace Manufacturing Co., Ltd, China) was used to melt Mg alloys. The Ar gas was pumped into the resistance furnace when the temperature of the furnace approached 673–773 K at a pressure of 1.5 MPa and flow rate of 5 l/min. After replacement of air with argon inside the furnace, the magnesium ingots were placed inside the furnace and the temperature was increased to 973–1003 K. Preheated and dried Sn metal, Mg-25La (wt.%) alloy, and Mn were added to the molten mixture, and the temperature was increased and held at 1023 K for 20 min. Hexachloroethane was then used for degassing and skimming of slag. Finally, Mg-3Sn-1Mn-*x*La (*x* = 0.2, 0.6, 1.0 and 1.4 wt.%) alloy plates were prepared by casting under Ar-shielded atmosphere by continuous rheo-rolling at 943 K with a roller speed of 0.052 m·s^−1^, and a flow rate of 15 l/min for cooling water^20^.

Samples of Mg-3Sn-1Mn-*x*La (*x* = 0.2, 0.6, 1.0 and 1.4 wt.%) alloys of dimensions of 15 mm × 15 mm × 10 mm and polished using standard metallographic procedure. X-ray diffraction (XRD; X’Pert, PANalytical B.V., Almelo, Holland) was used to analyze precipitates in the Mg-3Sn-1Mn-*x*La (*x* = 0.2, 0.6, 1.0 and 1.4 wt.%) alloys. Samples with different La concentrations were first polished and etched using a solution consisting of 13 vol.% HCl, 47 vol.% C_2_H_5_OH, and 40 vol.% H_2_O at room temperature for 0.2 s, followed by scanning electron microscopy (SEM; SSX-550, Shimadzu, Kyoto, Japan) to study the distribution of each element in the matrix. Samples with different La concentrations were processed into Φ 3 mm × 0.5 mm discs using a spark-cutting machine (DK7740, Precision Machinery Co., Ltd, China); the discs were then ground to a thickness of 80 μm and further thinned using a precision ion polishing System (Gatan 691, USA). The microstructure and precipitate phase in the alloy was analyzed using high-resolution transmission electron microscopy (HRTEM, Tecnai G^2^ F20, FEI, Oregon, USA). Uniaxial tensile tests were performed on a MTS 810 mechanical properties testing system (MTS, USA) at a constant strain rate of 5 × 10^−3^ s^−1^ at room temperature. A MTS LX300 laser extensometer was used to calibrate and measure the sample strain on tensile loading. For each condition, three tests were performed to obtain the average mechanical property data. After tensile tests, the alloy with the best mechanical properties was selected to study the deformed microstructure by HRTEM. The optimal Mg-3Sn-1Mn-*x*La (wt.%) alloy plate was deformed to 2% elongation, and specimens were cut from the gage of the deformed samples, to fundamentally understand the impact of precipitate phase on dislocation movement via HRTEM.

## Results

### Microstructure of Mg-3Sn-1Mn-xLa alloys

**Figure 2:**
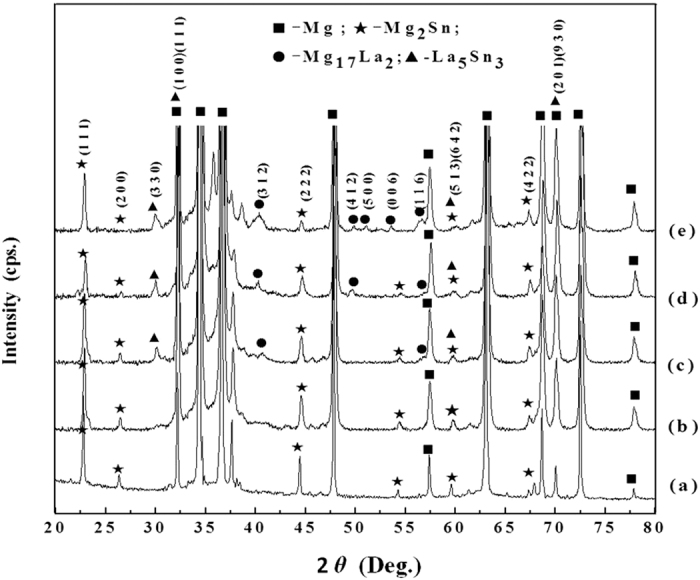
XRD patterns for the Mg-3Sn-1Mn-*x*La alloys with different La addition: (**a**) 0.0 La; (**b**) 0.2 La; (**c**) 0.6 La; (**d**) 1.0 La; (**e**) 1.4 La. Each phase indicated by different symbols.

Figure 2 shows the effect of La concentration on precipitate formation in Mg-3Sn-1Mn-*x*La (wt.%) alloy plates as studied by X-ray diffraction for different La concentrations. It can be seen that α-Mg and Mg_2_Sn were the two main phases in Mg-3Sn-1Mn-0.2La (wt.%) alloy (curve (b)). However, the diffraction peaks of Mg_2_Sn phase at (111), (200), (222) and (513) were weaker than those observed in Mg-3Sn-1Mn (wt.%) alloy. As shown in curve (c) in Fig. 2, when the La concentration was increased to 0.6 wt.%, diffraction peak intensities associated with the Mg_2_Sn phase at (111), (200), (222) and (513) were further decreased. The Mg_17_La_2_ phase emerged at (312) and (116) and the La_5_Sn_3_ phase emerged at (330). The diffraction peaks corresponding to La_5_Sn_3_ phase at (111) and (930) overlapped with the α-Mg phase at (100) and (201), respectively, while the diffraction peak at (642) overlapped with the diffraction peaks of Mg_2_Sn phase at (513). As the La concentration was increased to 1.0 wt.% (curve (d)), the diffraction peak intensities corresponding to Mg_2_Sn phase at (111), (200), (222) and (513) was further decreased. A new diffraction peak emerged at (412) position and was identified as Mg_17_La_2_ phase, suggesting a gradual increase in the volume fraction of Mg_17_La_2_ phase. Meanwhile, the diffraction peak intensity at (330) was also increased for the La_5_Sn_3_ phase. As the La concentration was increased to 1.4 wt.%, the diffraction peak intensities of the Mg_2_Sn phase at (200), (222) and (513) were very weak as shown in curve (e) in Fig. 2. The intensities were increased for Mg_17_La_2_ phase ((312) and (116) peaks) and La_5_Sn_3_ phase ((330) peak). New diffraction peaks emerged at (500) and (006) were identified as Mg_17_La_2_ phase.

**Figure 3:**
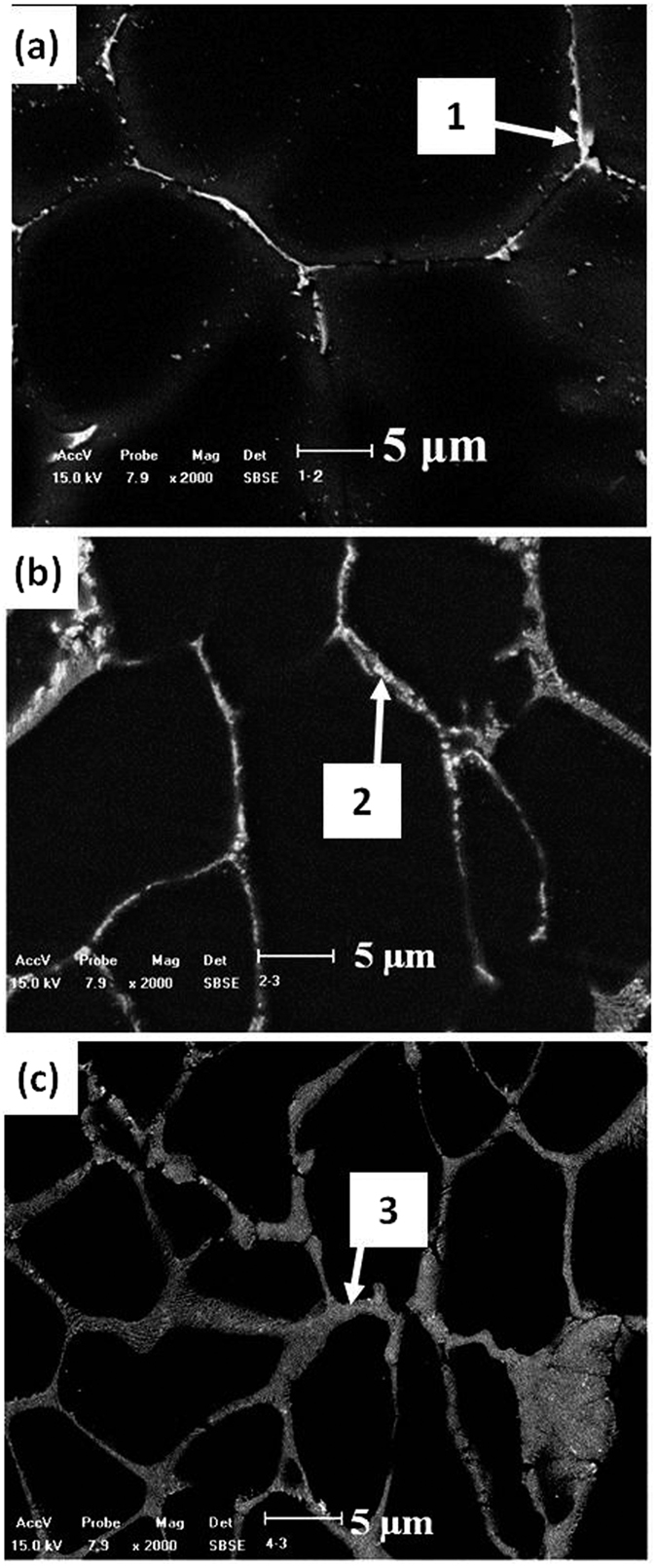
SEM observations show the morphologies of (**a**) Mg-3Sn-1Mn-0.2La, (**b**) Mg-3Sn-1Mn-0.6La and (**c**) Mg-3Sn-1Mn-1.4La alloys. Chemical compositions of regions 1, 2 and 3 detected by EDS and listed in Table 1.

**Figure 4:**
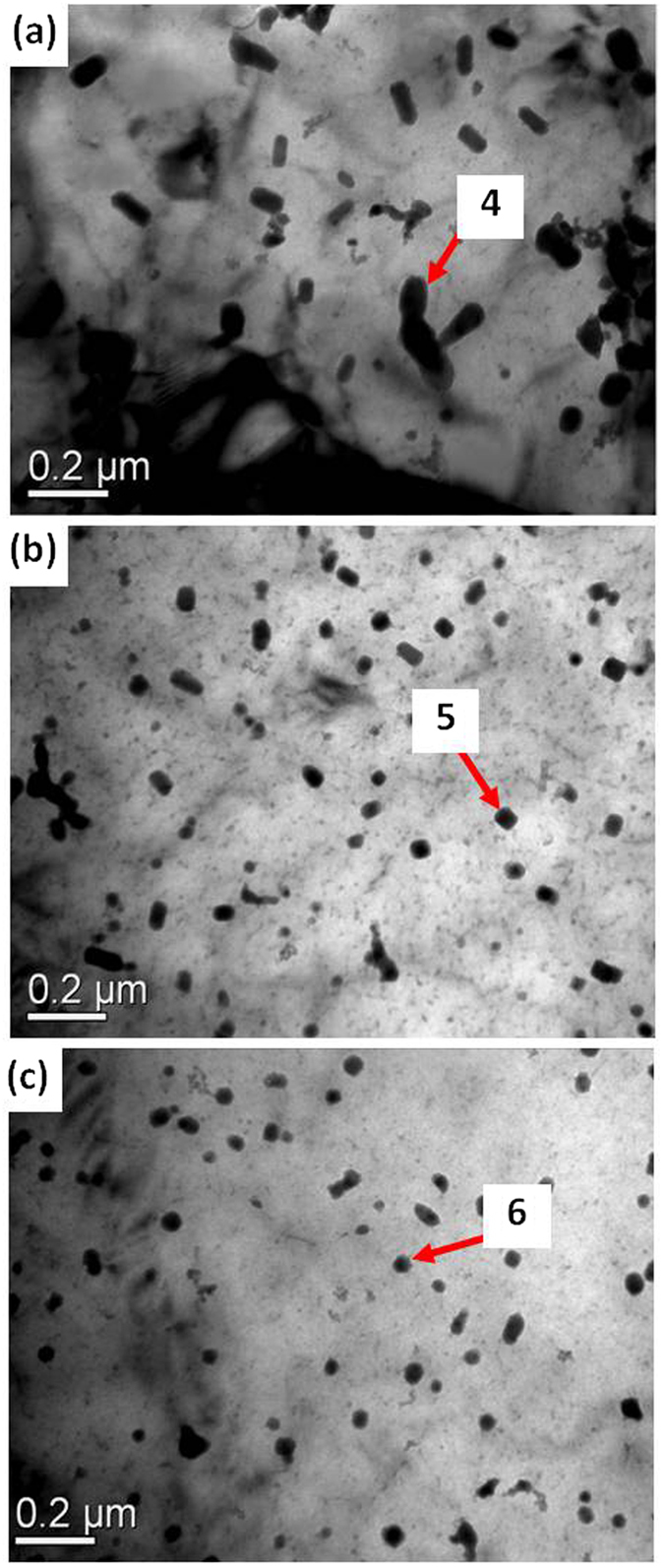
TEM images show the morphologies of (**a**) Mg-3Sn-1Mn-0.2La, (**b**) Mg-3Sn-1Mn-1.0La and (**c**) Mg-3Sn-1Mn-1.4La alloys observed along [0001]_Mg_ zonal axis. Chemical compositions of regions 4, 5 and 6 by EDS are listed in Table 1.

Figure 3a–c are SEM micrographs and the associated results of energy dispersive X-ray spectrometer (EDS) analysis of α-Mg grain boundaries, labeled as 1–3 in Table 1. These results indicate that in Mg-3Sn-1Mn-0.2La (wt.%) alloy, the compounds formed at the grain boundaries were primarily composed of Mg and Sn with a ratio of ~2:1, based on atomic %. Based on XRD and EDS analyses, the compound formed at α-Mg grain boundaries was determined to be Mg_2_Sn phase, which was confirmed by TEM studies (Fig. 4a–c). The associated results of energy dispersive X-ray spectrometer (EDS) analysis at the α-Mg grain boundaries are labeled as 4–6 (Table 1). Only a short, rod-like Mg_2_Sn phase was observed, with an average length of 100 ± 5 nm and an average width of 45 ± 2 nm. XRD and EDS results indicated that no La-containing phase precipitated in the Mg-3Sn-1Mn-0.2La (wt.%) alloy, and majority of La was present in solid solution in the α-Mg matrix.

**Figure 5:**
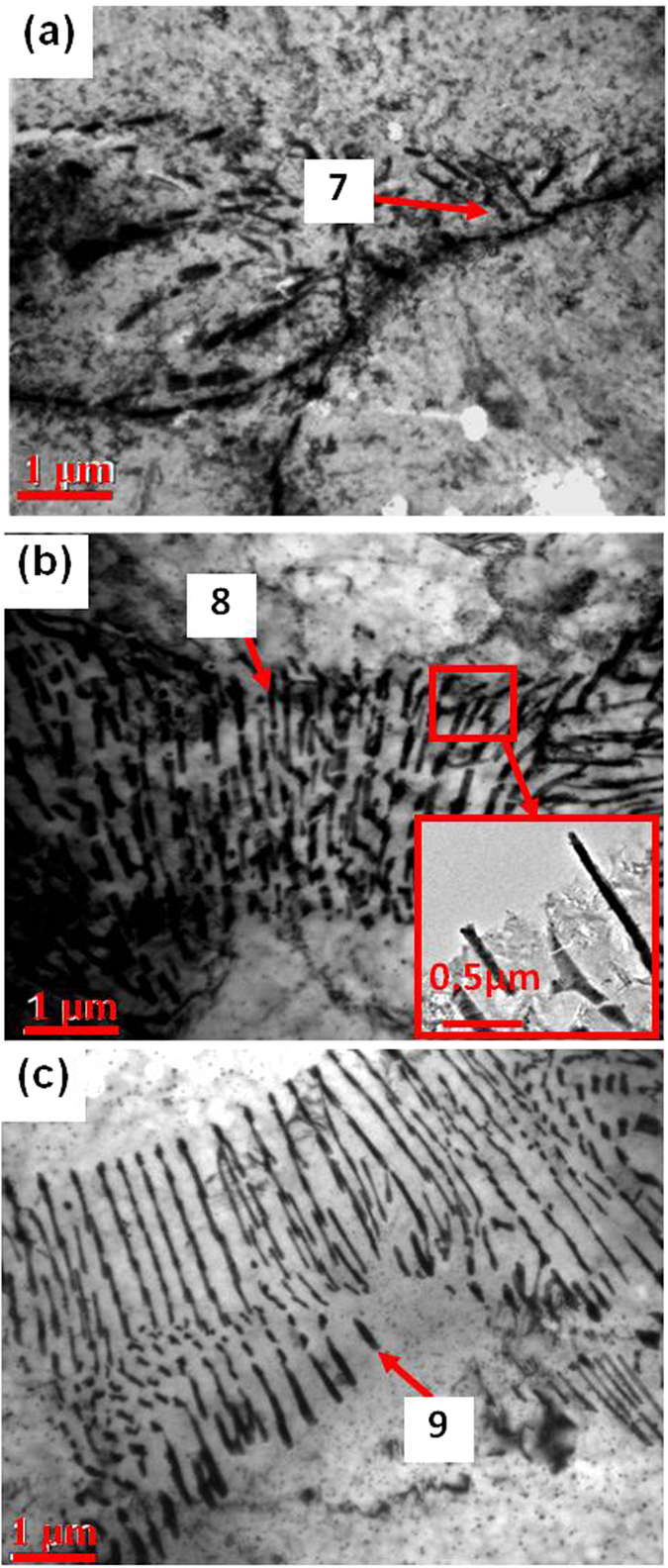
TEM images show the morphologies of (**a**) Mg-3Sn-1Mn-0.6La, (**b**) Mg-3Sn-1Mn-1.0La and (**c**) Mg-3Sn-1Mn-1.4La alloys close to grain boundaries. The incideent beam was aligned to [0001] zonal axis of α-Mg matrix. Chemical compositions of regions 7, 8 and 9 by EDS are listed in Table 1.

**Figure 6:**
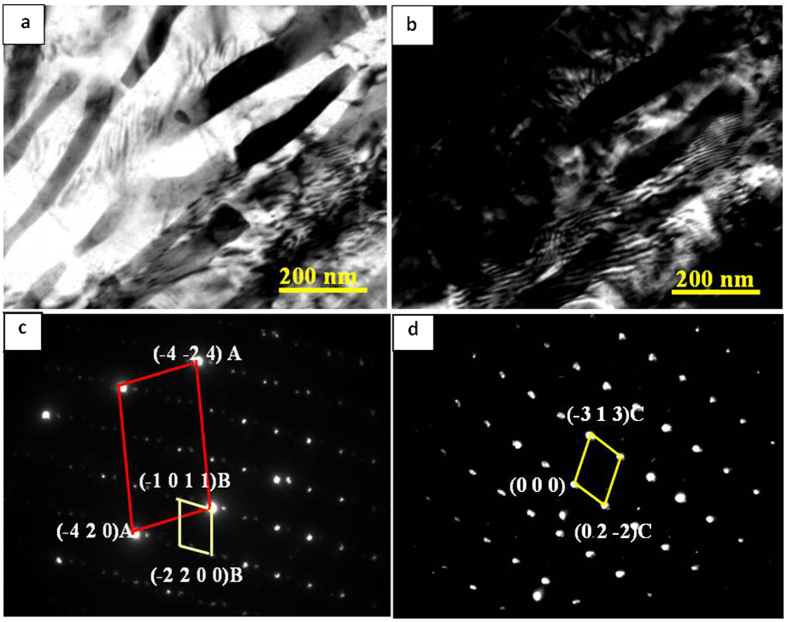
(**a**) Bright field and (**b**) dark field TEM images as well as diffraction patterns (**c,d**) of plate-shaped compound.

EDS analysis revealed that the chemical composition was 56.5Mg-26.5Sn-17.0La (wt.%) for the plate-shaped compound at the α-Mg grain boundary of Mg-3Sn-1Mn-0.6La alloy (No. 3 in Table 1). The TEM observations, suggested that this new phase was present as plate-like at α-Mg grain boundary, with an average length of 380 ± 10 nm and an average width of 110 ± 5 nm (Fig. 5a). This plate-shaped compound had a Mg:Sn:La ratio of ~66:26:7 (at.%), as determined from EDS analysis (No. 7 in Table 1). As shown in Fig. 5b, the average length of the plate-shaped compound at the α-Mg grain boundary of Mg-3Sn-1Mn-1.0La (wt.%) plates approached ~560 ± 20 nm and an average width of 90 ± 5 nm, indicating a significant increase in volume fraction, and the compound appeared to be evenly distributed at the α-Mg grain boundary. The related EDS analysis is listed in Table 1 (labeled as 8). This plate-shaped compound grew perpendicular to the α-Mg grain boundary and extended toward the inside of the matrix, preventing the α-Mg grain boundary from sliding (Figs 5 and 6), and thereby potentially increases the strength of the alloy. In addition, the refinement of the Mg_2_Sn phase was beneficial in improving the mechanical properties of Mg-Sn-Mn alloy^17^. This aspect is supported by the observation that a spherodized Mg_2_Sn phase of ~50 ± 2 nm in diameter was formed and evenly distributed between the plate-shaped compounds (the inset in Fig. 5b). The volume fraction of compound increased in comparison to the other two alloys (Fig. 3 and No. 3 in Table 1). The Mg_2_Sn phase continued to be intragranular precipitate, and variation in morphology and distribution were not significant, as shown in Fig. 4c. However, the quantity of the plate-shaped compounds at the α-Mg grain boundary was increased. The average length increased to 2300 ± 50 nm, whilst the average width decreased to 70 ± 5 nm (Fig. 5c).

Figure 6a,b are the morphologies of the plate-shaped compound observed along the zonal direction of [10,11]_Mg_, and Fig. 6c,d show the diffraction patterns of these plate-shaped compounds that overlapped with the surrounding α-Mg matrix. Two systems of periodic spots can be seen in Fig. 6c. One set of spots indicated by red lines reveal R_11_ = 3.5, R_12_ = 7.4, along with an angle of 74° between R_11_ and R_12_. Therefore, these spots are indexed as La_5_Sn_3_ phase (body-centered tetragonal, BCT, *a* = *b* = 1.2740 nm, *c* = 0.6343 nm) according to the [122]_La5Sn3_ zone axis. The other set of spots indicated by yellow lines reveal R_21_ = 1.5, R_22_ = 2.2, and an angle of 67° between R_21_ and R_22_. These spots are indexed according to the [11–23]_Mg17La2_ zone axis of the Mg_17_La_2_ (HCP, *a* = *b* = 1.0360 nm, *c* = 1.0240 nm) phase (Fig. 6c). Moreover, another set of relatively regular spots were observed in Fig. 6d, showing R_31_ = 1.6, R_32_ = 2.1 as well as an angle of 106° between R_31_ and R_32_. These spots are indexed as Mg_2_Sn (face-centered cubic, FCC, *a* = *b* = *c* = 0.6759 nm) phase according to the [433]_Mg2Sn_ zone axis. Thus, it was confirmed that this plate-shaped compound was composed of La_5_Sn_3_, Mg_2_Sn and Mg_17_La_2_ phases, which is consistent with the XRD analysis presented in Fig. 2. This new-type of composite structure was first observed in this study and has not been previously reported^8^,^9^,^10^,^11^,^12^,^13^.

**Figure 7:**
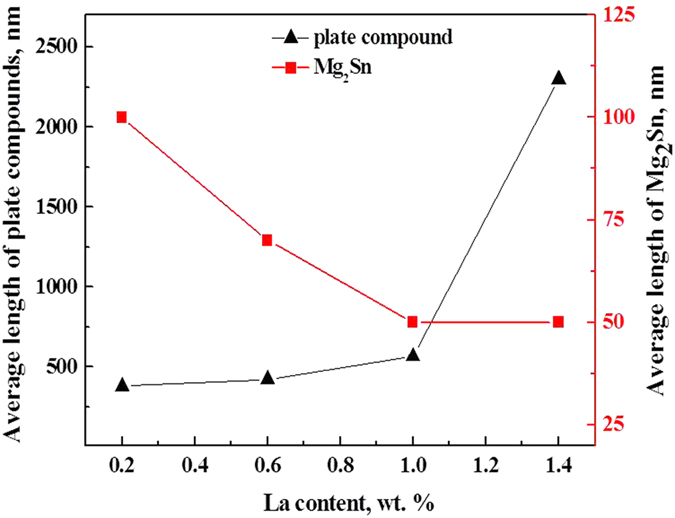
Effect of La concentration on the type and size of precipitates in Mg-3Sn-1Mn-xLa (wt.%) alloys.

With increasing La concentration in the range of 0.2–1.4 wt.%, the intragranular Mg_2_Sn phase was gradually refined, spherodized, and uniformly distributed. The average length of the plate-shaped compound (La_5_Sn_3_ and Mg_17_La_2_ phases) was gradually increased, accompanied by decreasing width and average length of Mg_2_Sn phase (Fig. 7).

### Mechanical properties of Mg-3Sn-1Mn-xLa alloys

**Figure 8:**
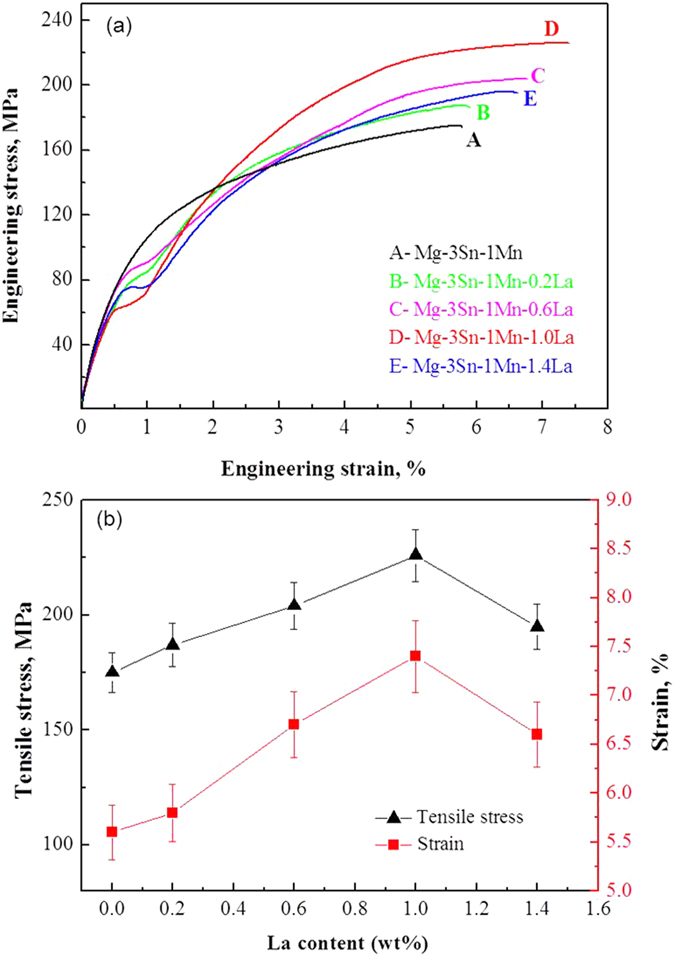
(**a**) Engineering stress-strain curves of Mg-3Sn-1Mn-*x*La alloys at room temperature. (**b**) Evolution of tensile stress and strain with the variation of La concentrations in Mg-3Sn-1Mn-*x*La alloys.

Engineering stress-strain plots of Mg-3Sn-1Mn-*x*La (*x* = 0.2, 0.6, 1.0 and 1.4 wt.%) alloys tested at room temperature are presented in Fig. 8a. The Mg-3Sn-1Mn (wt.%) alloy showed no yield plateau during tensile straining. However, on the addition of La, yield plateau was observed for Mg-3Sn-1Mn-*x*La (wt.%) alloys during tensile tests, which is different from the deformation characteristics of the reported Mg-alloys^4^. Figure 8b summaries the influence of La concentration on tensile stress and elongation of Mg-3Sn-1Mn-*x*La (wt.%) alloys. With increased La concentration, the tensile strength and elongation of the alloys was first increased and then decreased, the peak values were achieved at 1.0 wt.% La. The Mg-3Sn-1Mn-1.0La (wt.%) alloy plate exhibited a tensile strength of 230 ± 10 MPa and elongation of 7.5 ± 0.2%. These values are 29% and 32% higher than the tensile strength (175 ± 5 MPa) and elongation (5.6 ± 0.1%) of Mg-3Sn-1Mn (wt.%) alloy, and are respectively 37% and 89% higher than the tensile strength and elongation of Mg-3Sn-1Mn-0.87Ce (wt.%) alloy^13^. The small but consistently observe improvement in elongation of ~2% is encouraging because Mg element is an intrinsic brittle metal. The symmetry of the hexagonal close-packed (HCP) crystal structure has limited number of independent slip systems, resulting in poor ductility at room temperature^21^. Moreover, the melting points of precipitates are higher than pure Mg. For example, La_5_Sn_3_such has a melting point of 1500 °C, whilst that of Mg_17_La_2_ is 672 °C. Thus, the stability of mechanical properties can be improved by the addition of La to Mg alloys.

## Discussion

### Mechanism of microstructure formation

The difference in electronegativity of alloying elements determines their ability to form compounds. The greater the difference in electronegativity, the larger the binding force between these elements, thus making them more likely to form a compound^13^,^22^. Table 2 summarizes the electronegativity difference between Mg, Sn, La and Mn. The maximum difference in electronegativity is between La and Sn, followed by Mg and Sn^13^,^22^. The solid solubility of La in the α-Mg matrix is 0.14 wt.%^23^, and thus, majority of La is dissolved within the α-Mg matrix, when 0.2 wt.% La was added to the alloy. In the Mg-3Sn-1Mn-0.2La (wt.%) alloy, La-containing phases did not form because of the fast cooling rate of the vibration plate during continuous rheo-rolling and the rapid solidification rate of the alloy^20^. At room temperature, the solid solubility of Sn in α-Mg matrix is 0.17 wt.%, and there was relatively more Sn in Mg-3Sn-1Mn-*x*La (wt.%) alloys, which was beneficial for the formation of the Mg_2_Sn phase. Zhou *et al*. calculated that the heat needed for Mg_2_Sn formation and the binding energy for the Mg_2_Sn phase was −20.82 kJ/mol and 251.83 kJ/mol, respectively, demonstrating the stability of Mg_2_Sn phase^24^. Hence, the Mg-3Sn-1Mn-0.2La (wt.%) alloy was primarily composed of α-Mg and Mg_2_Sn phases. La and Sn combined with each other (Table 2), according to the qualitative thermokinetic criteria theory suggested by Mendis *et al*.^25^.

**Figure 9:**
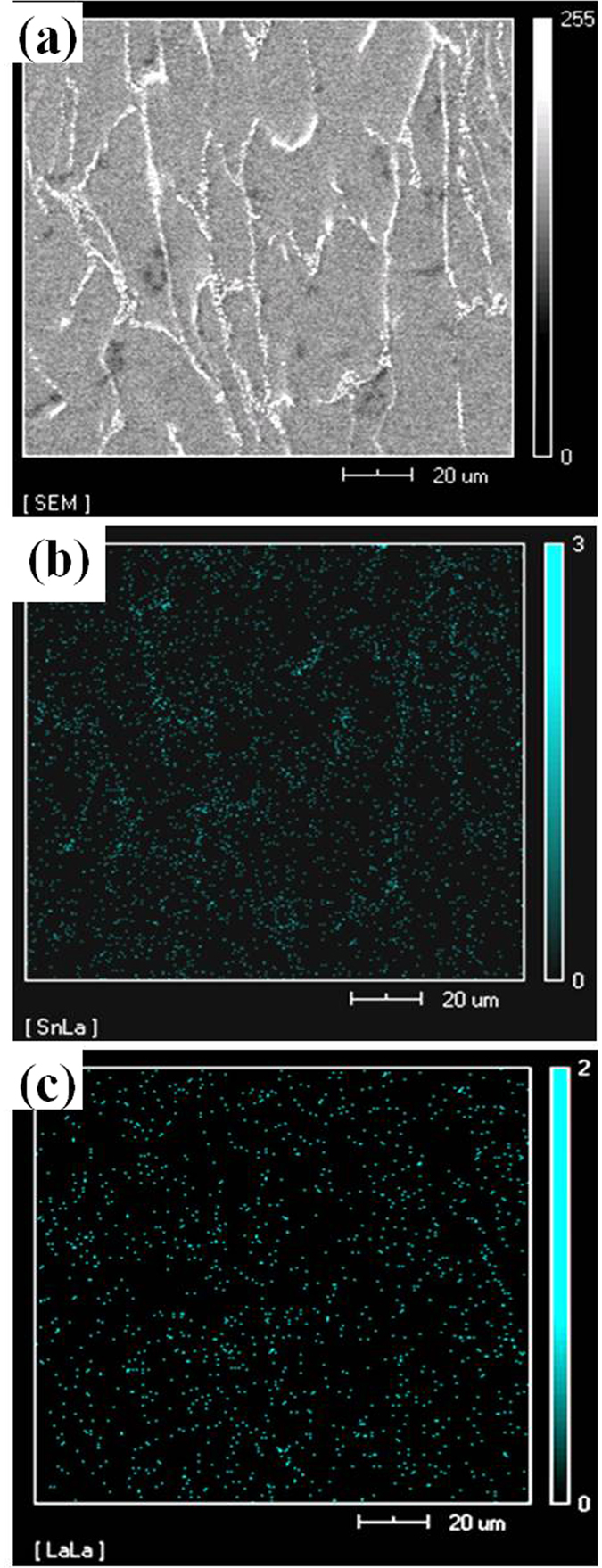
SEM image (**a**), Sn (**b**) and La (**c**) distribution maps of Mg-3Sn-1Mn-1.0La (wt.%) alloys.

In addition to the microalloying ability, La can also facilitate the nucleation of Sn-containing phases^25^. Thus, with increase in La concentration, a significant amount of La was homogeneously distributed within the matrix, as indicated by SEM observations, which facilitates an uniform distribution of Sn phase near the grain boundaries and the nucleation of Mg_2_Sn phase (Fig. 9a–c). During the growth of Mg_2_Sn phase, the surrounding La atoms segregate on the surface of initial Mg_2_Sn phase, thus increasing the surface energy and making it very difficult for surface atoms to continuously diffuse into the inner Mg_2_Sn phase, which hinders further growth of Mg_2_Sn phase, resulting in the gradual refinement and spherodization of Mg_2_Sn phase. As a result, the Mg_2_Sn phase becomes smaller and the aspect ratio is close to 1 with increase in La concentration (Fig. 4).

**Figure 10:**
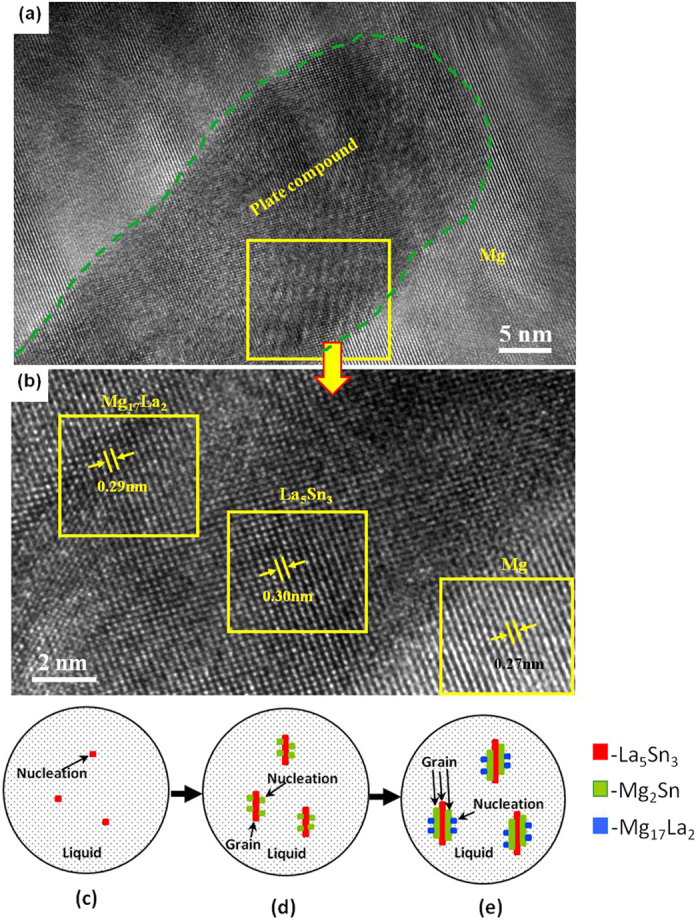
High-resolution TEM images (**a,b**) show the morphologies of the plate compound and α-Mg matrix. Schematic diagram of plate-shaped compound formation: (**c**) La_5_Sn_3_ phase nucleation; (**d**) Mg_2_Sn phase adheres to La_5_Sn_3_ phase for nucleation; (**e**) Mg_17_La_2_ adheres to Mg2Sn for nucleation.

During the solidification of the alloy, as the solute concentration was high to form intermetallic compounds, the phase with the highest melting point will be the first to form. Then, as the temperature of the molten alloy decreases, eutectic reactions occur among the remaining alloying elements and phases with low melting point start to form^26^. Because of the large electronegativity difference between La and Sn (Table 2), and higher melting point of La_5_Sn_3_ phase (1773 K) than Mg_2_Sn (1043 K) and Mg_17_La_2_ phases (945 K), La_5_Sn_3_ phase first nucleated and grew at the α-Mg grain boundary, when the La concentration was 0.6 wt.% (Fig. 10a,b). Idbenali *et al*. showed that La_5_Sn_3_ phase was stable at room temperature^27^. The La_5_Sn_3_ phase contained few Sn atoms and no Mg atoms and grew perpendicular to the α-Mg grain boundary, resulting from eutectic transformation during the final solidification. Thus, during the formation of La_5_Sn_3_ phase at the α-Mg grain boundary, Mg and Sn atoms were constantly expelled and accumulated at the solidification interface, such that the nucleation of Mg_2_Sn phase occurred. The formation of La_5_Sn_3_ phase provided a site for the formation of Mg_2_Sn phase (Fig. 10c). As the Mg_2_Sn phase adhered to the surface of the La_5_Sn_3_ phase and nucleation started, its surface energy was decreased; therefore, nucleation occurred at lower degree of undercooling (Fig. 10d). Additionally, since the electronegativity difference between Mg and Sn is greater than between Mg and La, and the melting point of the Mg_2_Sn phase is higher than Mg_17_La_2_ phase, thus, the Mg_2_Sn phase nucleated on La_5_Sn_3_ phase, prior to the nucleation of Mg_17_La_2_ phase. With nucleation and growth of Mg_2_Sn phase, Mg and La atoms were expelled and accumulated at the liquid-solid interface, providing constituents for the formation of the Mg_17_La_2_ phase, as well as the adherent points for nucleation of the Mg_17_La_2_ phase, thus facilitating the formation of the Mg_17_La_2_ phase (Fig. 10e). Therefore, at the α-Mg grain boundary, La_5_Sn_3_, Mg_2_Sn and Mg_17_La_2_ phases nucleated and grew alternately, forming a plate-shaped compound comprising of three phases, confirmed by HRTEM (Fig. 10) and XRD and EDS analyses (Fig. 2 and Table 1).

As the La concentration increased, more La_5_Sn_3_ and Mg_17_La_2_ were gradually formed and plate-shaped compounds comprising of La_5_Sn_3_, Mg_2_Sn, and Mg_17_La_2_ phases at the α-Mg grain boundary. In Mg-La alloys, higher concentration of La increases the cohesive energy of Mg-La compound and α-Mg grain stability, which is favorable for increasing the stability of La-containing phases^28^. Thus, the stability of La_5_Sn_3_ and Mg_17_La_2_ phases increased with increasing La concentration, promoting increase in La_5_Sn_3_ phase. The formation of La_5_Sn_3_ phase consumed majority of Sn atoms in the liquid phase, resulting in gradual decrease in Mg_2_Sn concentration.

### Effects of microstructure on mechanical properties of alloys

It can be seen from Fig. 8 that after the addition of La, the Mg-3Sn-1Mn-xLa alloy showed a yield plateau during stretching, which is seldom observed in Mg alloys^4^. The yield plateau is mainly due to the pinning effect of the precipitates such as La_5_Sn_3_, Mg_2_Sn, and Mg_17_La_2_ on dislocations. As dislocations are pinned by the precipitates, higher stress is required for dislocations to escape the blockage of the precipitates and move, thus generating the upper yield point. Once dislocations eventually overcome the pinning force, they move and the stress decreases, generating the lower yield point. Several studies have shown that rare earth atoms can effectively impede dislocation movement^4^,^29^,^30^.

**Figure 11:**
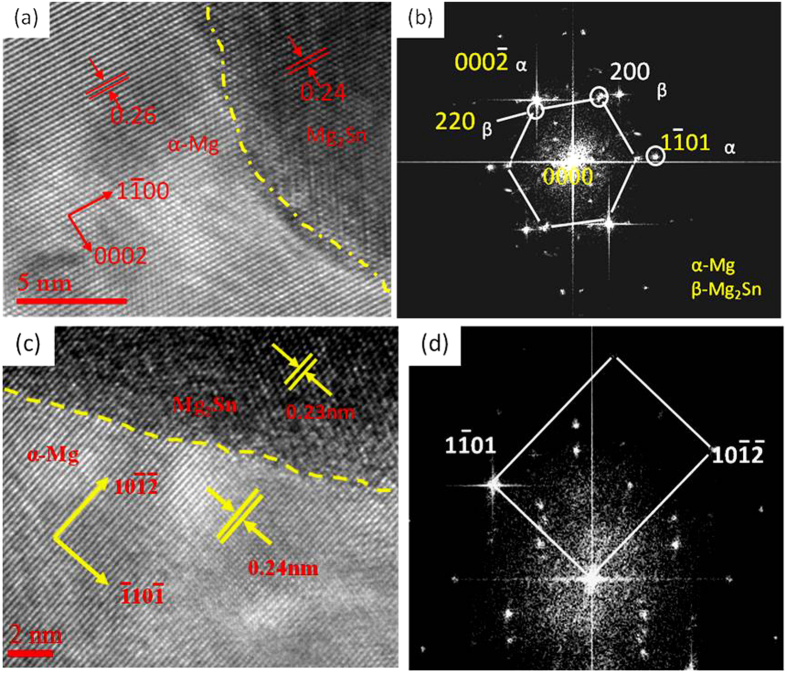
High-resolution TEM images (**a,b**) show the morphologies of the plate compound and α-Mg matrix. The corresponding diffractogram (**c,d**) obtained by fast Fourier Transform (FFT).

In Mg-3Sn-1Mn-*x*La (*x* = 0.2, 0.6, 1.0 and 1.4 wt.%) alloys, the dimension, size and distribution of Mg_2_Sn phase plays an important role in dictating the ultimate mechanical properties of the alloy^17^,^31^. Figure 11 shows a HRTEM image and Fourier transformation of Mg_2_Sn phase and α-Mg matrix. The Mg_2_Sn phase were coherent with α-Mg matrix along the [1–100]_Mg_ direction, with an interplanar spacing of 0.26 nm for the [000–2]_Mg_ plane of α-Mg matrix and 0.24 nm for the [220]_Mg2Sn_ plane of Mg_2_Sn phase. The lattice mismatch between the Mg_2_Sn phase and α-Mg matrix along with the resulting stress field can influence the deformation of alloy by restricting their dislocation movement. The degree of mismatch is defined by the following equation^32^,^33^:


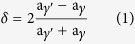


where a_γ_ and a_γ′_ are to the interplanar spacing of γ and γ′ phase, respectively. The Mg_2_Sn phase and α-Mg matrix have a small degree (8.0%) of mismatch and low interfacial strain energy between (220)_Mg2Sn_ and (0002)_Mg_ lattices. Their interface is relatively stable and less likely to allow nucleation or initiation of microcracks during deformation, suggesting that the Mg_2_Sn phase is helpful in improving the mechanical properties of Mg-3Sn-1Mn-*x*La (*x* = 0.2, 0.6, 1.0 and 1.4 wt.%) alloys. Additionally, there is an angle of 5.5° between the (220)_Mg2Sn_ plane of the Mg_2_Sn phase and the (0001)_Mg_ plane of the α-Mg matrix. Since the (0001)_Mg_ plane is the glide plane of dislocations in Mg alloys^34^, the formation of Mg_2_Sn phase can effectively impede dislocations from the basal slip, leading to an increase in the strength of the alloys.

**Figure 12:**
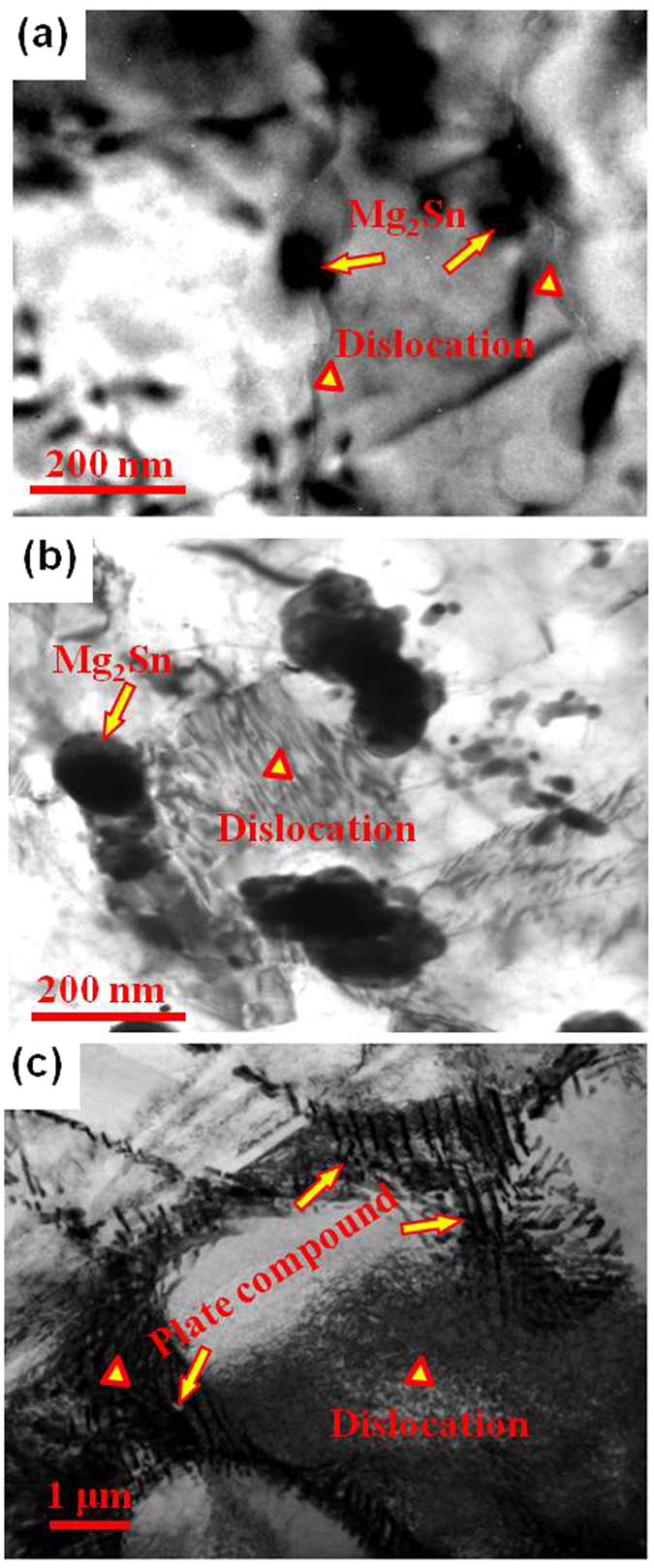
TEM images reveal that the pile-up effect of precipitates to dislocations in the Mg-3Sn-1Mn-1.0La (wt.%) alloy after tension with 2% engineering strain at room temperature. (**a,b**) show the pinned dislocations (triangle) by the spherical Mg2Sn phase, whilst (**c**) indicates high density of dislocations impeded by the platelike compound (arrows).

After deformation to a strain of 2%, a high density of dislocations can be observed around the Mg_2_Sn phase in the Mg-3Sn-1Mn-1.0La alloy (Fig. 11), suggesting that the Mg_2_Sn phase had a pinning effect on the movement of dislocations. In fact, it is reported that the Mg_2_Sn phase is a hard phase in Mg-3Sn-1Mn-*x*La (*x* = 0.2, 0.6, 1.0 and 1.4 wt.%) alloys with a microhardness of 1.19 GPa^7^,^8^,^9^. When the dislocations move to the region near the Mg_2_Sn particles, they indicated a tendency to bypass the Mg_2_Sn phase (Fig. 12a), leading to Orowan strengthening^35^. A similar scenario has been reported by Sasaki *et al*.^36^. On the other hand, the Mg_2_Sn phase was gradually refined, spherodized, and more uniformly distributed with increased La concentration (Figs 4 and 7), leading to increase in the pinning effect of Mg_2_Sn phase on dislocations slip^17^. For example, the length of Mg_2_Sn phase decreased from 100 nm to 50 nm with increased La addition from 0.2% to 1.4%, exhibiting equiaxial morphology.

Based on the well-known Orowan mechanism, the strength increment due to precipitation strengthening, *σ*_prec._, is given by equation (2)^35^:


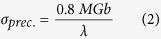


where *M* is the Taylor factor, *G* is the shear modulus, *b* is the Burgers vector and *λ* is the average spacing between neighboring particles. Apparently, a decrease in both particle size and the spacing of adjacent precipitates not only increases the stress required for dislocation movement, but also dislocation density, leading to increased strength of the studied alloys. With increase in La concentration to 1.0 wt.%, a plate-shaped compound composed of La_5_Sn_3_, Mg_2_Sn and Mg_17_La_2_ phases formed at the α-Mg grain boundary of the Mg-3Sn-1Mn-1.0La (wt.%) alloy. Despite increased average length to 560 ± 10 nm, the width of the plate compounds reduced to 90 ± 5 nm, accompanied by a decrease in length of Mg_2_Sn particles to 50 nm. Following equation (2), the strength associated with precipitation strengthening should increase because of reduced spacings between plate-shaped compounds and spherical Mg_2_Sn particles. This deduction was supported by tensile tests, as indicated in Fig. 8. Grain boundary sliding plays a significant role during the plastic deformation of the alloy, therefore, the morphology of the precipitates at α-Mg grain boundary and the energy of α-Mg grain boundary are important factors in governing the strain of the alloy^37^. The plate-shaped composite was characterized by HRTEM (Figs 10 and 11) and the degree of mismatch was 4.6% according to equation (1). This value means that the interface has a lower strain energy and this kind of interface is more stable, less likely to initiate nucleation and growth of microcracks during deformation. Moreover, this plate-shaped compound is formed near the α-Mg grain boundary and grows perpendicular to the boundary and toward inside of the α-Mg matrix, by pinning the α-Mg grain boundary and impede the sliding of α-Mg grain boundary (Figs 5, 6, 10 and 11). As the dislocations move to a nearby location in the vicinity of the plate-shaped compound, they pile up because of impeding effect of the compound (Fig. 12), leading to an increase in the strength of the alloy.

As the La concentration approached 1.4 wt.%, more plate-shaped compounds were formed and gradually became thinner, with average thickness of 70 ± 5 nm and significantly increased average length of 2300 ± 50 nm. According to equation (2), the stress increment derived from precipitation strengthening should increase because the thickness of plate-shaped compounds decreased (Figs 5c and 6). However, Fig. 8 shows that both tensile strength and strain decreased with increasing La content from 1.0% to 1.4%. The main reason must be related to the high degree of segregation of the plate compounds (Figs 3c and 5c), though the size of Mg_2_Sn particles still remained constant as small as ~50 nm, in comparison to that of Mg-3Sn-1Mn-1.0La (wt.%) alloy. As a result, the total pinning effect of the precipitates on the α-Mg grain boundary was decreased due to the high degree of segregation of plate-shaped compounds, despite similar dimension of Mg_2_Sn particles, leading to a relatively weak hardening effect compared to the alloy containing 1.0 wt.% La.

A power-law hardening equation can be used to describe the flow strength of metal materials, as follows^38^:


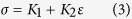


where *σ* is the flow stress, *ɛ* is the strain, n is the work hardening exponent, *K*_1_ represents the initial yield strength of the materials and *K*_2_ represents the increment in strength due to work hardening. According to equation (3), instability necking is activated, when *ɛ*_u_ = n (uniform elongation) during uniaxial tension of sheet specimens. Referring to Fig. 8a, it is evident that the Mg-3Sn-1Mn-1La alloy had the highest *n* value among all the five alloys. Thus, the decrease in strain of Mg-3Sn-1Mn-1.4La alloy must be associated with the localized necking resulted from the high degree of segregation of plate-shaped compounds.

## Conclusions

We have elucidated here the effect of La on the microstructure and properties of Mg-3Sn-1Mn-xLa (*x* = 0.2, 0.6, 1.0 and 1.4 wt.%) alloys produced by continuous rheo-rolling process. The major conclusions are as follows:

- (a) A maximum 0.2 wt.% La was completely dissolved in α-Mg matrix to form a solid solution with precipitated Mg_2_Sn phase, which was coherent with the α-Mg matrix along the [1–100]_Mg_ zonal direction. The (220) plane of the Mg_2_Sn phase and the (0001) plane of the α-Mg matrix formed a 5.5° angle. The Mg_2_Sn phase had a pinning effect on dislocation slip.
- (b) With increase in La content to 0.6 wt.%, the La_5_Sn_3,_ Mg_2_Sn and Mg_17_La_2_ phases nucleated alternately to form a new-type plate-shaped three-phase compound. This plate-shaped compound was coherent with α-Mg matrix along the [10–1–2]_Mg_ zonal direction, and grew perpendicular to the inside of α-Mg grain, producing pinning effect on the α-Mg grain boundaries and dislocations.
- (c) At La content in the range of 0.2–1.4 wt.%, the Mg_2_Sn phase was gradually refined and spherodized in α-Mg matrix. The length of the plate-shaped compound was gradually increased from 380 ± 10 nm to 2300 ± 50 nm, and the width reduced from 110 ± 5 nm to 70 ± 5 nm. The tensile strength and elongation of the alloy gradually increased with increase in La content from 0.2–1.0 wt.% and then decreased on further increase in La content from 1.0–1.4 wt.%, mainly associated with the segregation of plate-shaped compounds.
- (d) At La content of 1.0 wt.%, the tensile strength and elongation of the alloy attained maximum. In comparison with Mg-3Sn-1Mn (wt.%) alloy, the tensile strength and elongation increased by 29% and 32%, respectively. In comparison with the Mg–3Sn–1Mn-0.87 Ce (wt.%) alloy, the tensile strength and elongation increased by 37% and 89%, respectively. The nanosized Mg_2_Sn particles and plate-shaped compounds played an important role in improving the mechanical properties.

## Additional Information

**How to cite this article**: Guan, R.G. *et al*. Nanoscale precipitates strengthened lanthanum-bearing Mg-3Sn-1Mn alloys through continuous rheo-rolling. *Sci. Rep*. **6**, 23154; doi: 10.1038/srep23154 (2016).

## Figures and Tables

**Table 1:** Chemical compositions of different regions in the Mg-3Sn-1Mn-*x*La alloys obtained by EDS analysis.

No.	In alloy	Element	wt.%	at.%
1	Mg-3Sn-1Mn-0.2 La	Mg	31.7	65.1
		Sn	68.3	34.9
2	Mg-3Sn-1Mn-0.6 La	Mg	53.9	85.6
		Sn	33.9	11.0
		La	12.2	3.4
3	Mg-3Sn-1Mn-1.4 La	Mg	56.5	87.1
		Sn	26.5	8.4
		La	17.0	4.6
4	Mg-3Sn-1Mn-0.2 La	Mg	40.3	76.7
		Sn	59.7	23.3
5	Mg-3Sn-1Mn-0.2 La	Mg	49.5	82.7
		Sn	50.5	17.3
6	Mg-3Sn-1Mn-0.2 La	Mg	54. 6	85.4
		Sn	45.4	14.6
7	Mg-3Sn-1Mn-0.6 La	Mg	27.9	66.2
		Sn	54.4	26.4
		La	17.7	7.4
8	Mg-3Sn-1Mn-1.0 La	Mg	35.8	73.8
		Sn	47.9	20.3
		La	16.3	5.9
9	Mg-3Sn-1Mn-1.4 La	Mg	34.8	73.1
		Sn	48.1	20.7
		La	17.1	6.3

Numbers represent the different positions in Figs 3, 4 and 5, respectively.

**Table 2:**
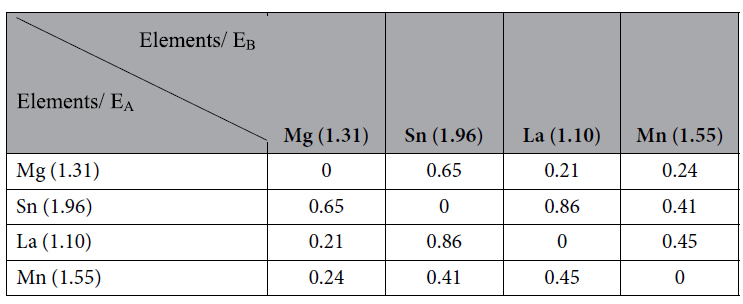
Differences of electronegativity value between Mg, Sn, La and Mn.
